# Cutaneous mucormycosis in the ICU

**DOI:** 10.1186/cc14168

**Published:** 2015-03-16

**Authors:** EH Herrero, M Sánchez, A Agrifoglio, L Cachafeiro, MJ Asensio, B Galván, A García de Lorenzo

**Affiliations:** 1 Hospital La Paz, Madrid, Spain

## Introduction

Mucormycosis is a devastating disease most commonly seen in immunosuppressed individuals. It has the propensity to disseminate in humans and cause rhinocerebral, pulmonary, gastrointestinal, and cutaneous infections. This study focuses on cutaneous mucormycosis, incidence, epidemiologic characteristics and mortality in intensive care medicine.

## Methods

We present a descriptive study in an ICU between the years 2001 and 2013 on the incidence of patients with cutaneous mucormycosis. Sociodemographics, comorbidities and laboratory data were recorded. Clinical data were collected to calculate the APACHE score. The main outcome was to analyze the epidemiological characteristics of patients with cutaneous mucormycosis and mortality.

## Results

Seven patients were identified with cutaneous mucormycosis between the years 2001 and 2013. The mean age of patients was 52 ± 4, with an APACHE score of 19 ± 9, and 57% died. All patients were admitted for trauma-related injury suffering blast, abrasive injuries or burns. Mortality among patients with signs of sepsis was 100%, and only in one of them was empirically antifungal therapy started; in the others antibiotic treatment was directed. Among patients without signs of sepsis, the survivor was treated with amputation where mucoral infection was isolated. Procalcitonin rose in all patients with signs of sepsis.

## Conclusion

Cutaneous mucormycosis is less common than other clinical forms, most frequently seen in inmunocompetent patients but potentially lethal if treatment is not rapid. Patients at risk are those with disruption of the normal protective cutaneous barrier. In these patients, if signs are of sepsis it is very important to suspect the possibility of infection by Mucor and initiate empiric antifungal treatment with surgery to avoid high mortality. Surprisingly, in our series, determination of procalcitonin showed high levels in spite of not having value in fungal infection.

